# How the General Public Navigates Health Misinformation on Social Media: Qualitative Study of Identification and Response Approaches

**DOI:** 10.2196/67464

**Published:** 2025-06-24

**Authors:** Sharmila Sathianathan, Adliah Mhd Ali, Wei Wen Chong

**Affiliations:** 1 Centre of Quality Management of Medicines, Faculty of Pharmacy, Universiti Kebangsaan Malaysia Kuala Lumpur Malaysia; 2 Pharmacy Department, Hospital Tengku Ampuan Rahimah Selangor Malaysia

**Keywords:** approaches, response, general public, health misinformation, social media

## Abstract

**Background:**

Social media is widely used by the general public as a source of health information because of its convenience. However, the increasing prevalence of health misinformation on social media is becoming a serious concern, and it remains unclear how the general public identifies and responds to it.

**Objective:**

This study aims to explore the approaches used by the general public for identifying and responding to health misinformation on social media.

**Methods:**

Semistructured interviews were conducted with 22 respondents from the Malaysian general public. The theory of motivated information management was used as a guiding framework for conducting the interviews. Audio-taped interviews were transcribed verbatim and imported into ATLAS.ti software for analysis. Themes were identified from the qualitative data using a thematic analysis method.

**Results:**

The 3 main themes identified were emotional responses and impacts of health misinformation, approaches used to identify health misinformation, and responses to health misinformation. The spread of health misinformation through social media platforms has caused uncertainty and triggered a range of emotional responses, including anxiety and feelings of vulnerability, among respondents who encountered it. The approaches to identifying health misinformation on social media included examining message characteristics and sources. Messages were deemed to be misinformation if they contradicted credible sources or exhibited illogical and exaggerated content. Respondents described multiple response approaches to health misinformation based on the situation. Verification was chosen if the information was deemed important, while misinformation was often ignored to avoid conflict. Respondents were compelled to take action if misinformation affected their family members, had been corrected by others, or if they were knowledgeable about the topic. Taking action involved correcting the misinformation and reporting the misinformation to relevant social media, enforcement authorities, and government bodies.

**Conclusions:**

This study highlights the factors and motivations influencing the general public’s identification and response to health misinformation on social media. Addressing the challenges of health misinformation identified in this study requires collaborative efforts from all stakeholders to reduce the spread of health misinformation and reduce the general public’s belief in it.

## Introduction

### Background

Social media has increasingly been used by the general public for health-related purposes, primarily for receiving social support and searching for and sharing health information [1]. Social media platforms offer unlimited access to prompt and easily accessible health information, making them a preferred channel for seeking health information [2]. The most commonly used social media platforms for this purpose include WhatsApp (WhatsApp LLC), YouTube (Google LLC), Facebook (Meta Platforms, Inc), and X (formerly known as Twitter; X Corp) [2,3]. Studies have shown that between 76% and 85% of respondents, including the general public and patients aged ≥18 years from Saudi Arabia and the United States, search for health information on social media [2,4].

Although social media may offer some advantages in the dissemination of health information, there is growing concern about the prevalence of health misinformation on these platforms. Health misinformation is defined as a health-related claim that is false or misleading because of a lack of supporting scientific evidence at a given time [5]. Studies have shown that the quality of health information on social media is generally poor, based on criteria such as content accuracy, information design, credibility, disclosure of user information, and interactivity [6]. A systematic review found that the 3 most common topics with health misinformation on social media were vaccines (32%), drugs and smoking issues (22%), and noncommunicable diseases (19%) [5].

Health misinformation can be harmful as it can cause unnecessary fear and lead the general public to make inappropriate decisions about their health, which can worsen physical health and even lead to increased morbidity and mortality [7-10]. One example is the widespread antivaccine content on social media that has contributed to decreases in vaccine acceptance and vaccination rates as well as an increase in preventable disease outbreaks [7]. Physicians have also expressed concerns about the dangers of health misinformation, describing regular encounters with patients who were hesitant to take potentially lifesaving medications or adhere to prescribed treatments owing to misinformation on the internet and social media [8]. Moreover, the narratives found in health misinformation often instill fear, anxiety, and mistrust in health institutions [9]. For example, health misinformation circulating on social media during the Ebola virus outbreak created hostility toward health care workers and contributed to challenges in efforts to control the epidemic [11]. In addition, misinformation may cause the general public to have reduced adherence to government public health policies, rendering these efforts ineffective [12-16].

Previous research has demonstrated that various factors can influence the perceived credibility of health misinformation on social media. These factors may collectively contribute to the rapid spread of misinformation, particularly during health crises such as the COVID-19 pandemic. One identified factor is the inclusion of claims of background evidence, such as an attached link or source, in misinformation messages, which can make messages appear more credible to the general public [17]. Social endorsements, such as a higher number of likes and shares, also enhance believability [18-20]. The perceived credibility of the message’s source is also important, with individuals tending to trust messages from respected authorities, close contacts, influencers, or celebrities, leading to further misinformation spread [20,21]. Health care professionals and organizations are often perceived as reliable sources of information; however, they are also exposed to misinformation on social media [17,22]. A qualitative study among physicians and nurses in the United States identified several cues for spotting health misinformation, including messages with exaggerated claims, grammar errors, unreliable links, and conspiracy content [22].

The general public’s response to health misinformation on social media, if they are able to identify it, has been shown to be influenced by their beliefs, cultural factors, and social media use. Beliefs in conspiracy theories, such as government secrets, and cultural beliefs, such as trust in traditional treatments over scientific evidence, contribute to the spread and acceptance of misinformation, as observed during the COVID-19 pandemic [23,24]. General public reactions to misinformation on social media also vary; some people choose to disregard the information [25], while others challenge and report it [26]. Fact-checking has been shown to reduce the spread of misinformation, but heavy reliance on social media as information sources reduces the critical verification process, leading to the further spread of health misinformation [27].

Due to the urgency of this issue, various measures have been proposed and adopted by organizations, governments, health care workers, and researchers to combat health misinformation on social media. These measures include regulating social media content, skillfully communicating public health messages, promoting verification and correcting health misinformation, warning about the sources of information, promoting evidence-based medicine, educating the general public about health information, conducting fact-checking efforts, improving critical thinking skills, and enhancing media and health literacy [10,28-31]. For example, the World Health Organization (WHO), recognizing the mounting concerns about health misinformation on social media, has adopted numerous measures to combat it, such as providing an avenue for reporting health misinformation, creating a MythBusters web page to debunk misinformation with facts and figures, and working toward amendments in social media policies [32,33]. While this is recognized as a pressing issue requiring a multidisciplinary effort, effectively addressing it requires a deeper understanding of how the general public identifies and responds to health misinformation on social media platforms. This understanding will enable the identification of areas requiring concerted efforts from all stakeholders to mitigate the spread of health misinformation and reduce the general public’s belief in it.

While previous studies on health misinformation have primarily focused on regions such as Europe, the Middle East, Africa, and parts of Asia, Southeast Asia remains underrepresented in this field of research [34,35]. Nevertheless, there has been notable rise in health misinformation on social media within the South East Asian region [35]*.* Given that culture plays a significant role in how health misinformation is perceived and managed [36-38], it is important to explore this issue within a Southeast Asian context. As a multicultural and multilingual country, Malaysia offers a unique setting for such studies [39]. The diverse cultural backgrounds and traditions within the Malaysian population can influence health information–seeking behaviors [40] and provide additional insights into health misinformation on social media. However, no studies have specifically examined how the Malaysian general public identifies and responds to health misinformation on social media. Therefore, this study aims to explore the approaches used by the Malaysian general public to recognize health misinformation on social media and their responses to it.

### Theory of Motivated Information Management

The theory of motivated information management (TMIM) serves as a guiding framework in this study. The TMIM explains how uncertainty influences information management behaviors [41] and has been widely applied to explore the barriers and motivations behind health information seeking [42-45]. While its application to health misinformation is relatively limited, it may offer valuable insights into how individuals engage with, process, and respond to health misinformation encountered on social media. It can also help explain how feelings of uncertainty drive health information–seeking behaviors in the context of health misinformation [46].

This theory is structured into 3 phases: interpretation, evaluation, and decision. In the context of health misinformation on social media, the interpretation phase occurs when individuals recognize discrepancies or uncertainties in health information or misinformation they encounter. This recognition may trigger emotional responses, such as anxiety, which can influence their approach to managing the information. Next is the evaluation phase, where individuals assess their ability to reduce uncertainty through health-related information seeking and consider the potential outcomes—both positive and negative—of obtaining new information. The likelihood of seeking information is influenced by individuals’ perceived ability to cope with the expected outcomes (coping efficacy), their ability to manage the communication process involved in seeking information (communication efficacy), and the perceived reliability of the information source (target efficacy). Finally, the decision phase determines the individuals’ course of action based on the evaluations from the previous phase, such as whether to search for additional health information or disregard the health misinformation encountered [41].

## Methods

### Study Design and Participants

This qualitative study involved individual semistructured interviews with members of the general public in Malaysia. This study design was selected to allow a deeper exploration of the factors influencing the management of health misinformation in this population, providing richer insights into the issue.

The respondents were initially recruited through advertisements posted on social media platforms, including Facebook, Instagram (Meta Platforms, Inc), WhatsApp, and X. Those who were interested in participating completed a Google Form providing their sociodemographic information and details about their social media activity. Potential respondents who met the inclusion criteria, which included being Malaysian, aged >18 years, and active social media users on the specified platforms, were then contacted. Individuals who were unable to participate in web-based interviews or could not speak English or Malay were excluded. For this study, active social media users were defined as individuals who engaged with content by liking, commenting, and sharing information on Facebook, Instagram, X, and WhatsApp. These social media platforms were selected because they are the most commonly used for seeking and sharing health information in Malaysia [47].

The respondents were then purposively selected by the researchers to ensure a diversity of sociodemographic characteristics, such as age, education, occupation, income, gender, state of residence, and religion. This was done to gather a broad range of views and perspectives on the issue of health misinformation in Malaysian social media and the responses toward it.

### Ethical Considerations

This study was approved by the Human Research Ethics Committee of Universiti Kebangsaan Malaysia (JEP-2022-037). Before conducting the interviews, the purpose of the interviews was explained to the participants, and their written informed consent was obtained. To ensure privacy and confidentiality, all transcripts were anonymized through pseudonymization. Each participant was assigned a unique identifier, and all potentially identifying information was removed during transcription. Only the research team had access to the anonymized data, which was stored on a secure, password-protected server. Following the interviews, RM 50 (US $11) was credited into the e-wallets of the respondents as compensation for their participation in this study.

### Data Collection

An interview guide was developed based on domains identified through literature review and guided by the TMIM framework, as outlined earlier [41]. Table 1 presents examples of interview questions aligned with the 3 key phases of the TMIM. In the interpretation phase, questions were designed to explore the types of health misinformation encountered on social media; the uncertainty recognized in the information; and the emotional responses triggered, such as anxiety. In the evaluation phase, interview questions focused on understanding how individuals identify health misinformation, their perceptions of social media as a source of health information, and their confidence in seeking accurate information and assessing its reliability. Finally, questions in the decision phase explored how individuals respond to health misinformation based on their evaluations, including disregarding the misinformation or using strategies for verifying health information. The interview guide was piloted with 2 respondents and amended to improve clarity and length. The pilot interviews were not included in the final analysis.

**Table 1:** Examples of questions used during the interview.

TMIM^a^ phases [41]	Description	Examples of questions
Interpretation	Individuals recognize discrepancies or uncertainties in health misinformation, which may trigger emotional responses.	Have you ever encountered information that conflicts with your beliefs? If so, what was the information? How did you feel upon encountering this (mis)information? What was your initial reaction? What do you think are the risks of health misinformation?
Evaluation	Individuals assess their ability to reduce uncertainty through health information seeking and evaluate potential outcomes.	What would you do when you are unsure about health information encountered on social media, and why? How did you determine whether it was health misinformation? How do you assess whether an information source on social media is credible or not? How confident are you in your evaluation?
Decision	Individuals decide on the course of action based on their evaluations.	What would you do if you discovered that health information encountered on social media is misinformation? How would you respond, and why? How would responding this way make you feel? Would you seek further information, and why?

^a^TMIM: theory of motivated information management.

The individual interviews were conducted online via Zoom (Zoom Video Communications) between June 2022 and February 2023. The interviews were conducted in English and Malay. Each interview session lasted between 35 and 62 minutes, with an average duration of 40 minutes. All interviews conducted were audio recorded with the respondents’ consent, and field notes were taken during the interview sessions. The audio recordings of the interviews were transcribed verbatim. Interviews conducted in Malay were first transcribed in Malay and then translated into English, with the translations reviewed by bilingual research team members. The transcripts were assigned a code, and potentially identifying information was removed. The transcripts were anonymized through a pseudonymization procedure. Data collection was conducted until data saturation was reached, which was determined by the repetition of themes and the absence of new insights. This was assessed collectively by the research team, who continuously compared data from interviews to identify recurring themes among respondents. Data collection continued until all emerging themes were fully explored and stopped when no new codes or themes emerged from the last 3 interviewees [48].

### Data Analysis

The transcripts were imported into ATLAS.ti (ATLAS.ti Scientific Software Development GmbH) to facilitate the coding process and identify themes from the qualitative data. The reflexive thematic analysis method by Braun and Clarke [49] was used, following 6 phases, which included familiarization with the data, identifying initial codes, searching for themes, reviewing themes, classifying themes, and generating reports. Initially, inductive coding was conducted independently by SS and WWC to derive preliminary codes from the data. These initial codes were subsequently reviewed, compared, and discussed in depth during multiple iterative meetings between the researchers to ensure rigor and consistency. Following this, abductive coding, guided by the TMIM framework, was incorporated to interpret and refine the codes and themes. Specifically, TMIM was applied to interpret and refine the categorization of themes by incorporating insights into how individuals manage uncertainty and seek or avoid information. This theoretical lens helped clarify the motivations behind participants’ behaviors, ensuring that the themes captured both empirical patterns and underlying motivational processes [50]. Final codes were determined through discussion-based consensus. Themes and subthemes were identified based on significant patterns observed in the data and were continuously revised and refined using the constant comparison approach [51]. Field notes were used to aid early analysis, and reflexivity was maintained by the researchers throughout the interviews and analysis phases via memos collected during and after the interviews [52]. All themes and subthemes were finalized through discussions among research team members until a consensus was achieved.

## Results

### Overview

A total of 22 respondents participated in this study. Respondents represented a diverse range of demographics, including age groups (ranging from 19 to 54 years), levels of education (ranging from secondary school to postgraduate degrees), incomes (ranging from <RM 1000 to >RM 6000), and professional backgrounds (including unemployed, self-employed, private sector, and government) and came from different states across Malaysia. The detailed characteristics of the respondents are included in Table 2.

**Table 2:** Respondents’ demographics.

Respondent number	Age	Sex	Education	Employment sector	Family income^a^
1	27	Female	First degree	Private sector	RM 5000-RM 6000
2	32	Male	First degree	Private sector	>RM 6000
3	54	Female	First degree	Government sector	>RM 6000
4	36	Female	Postgraduate	Government sector	>RM 6000
5	19	Female	Diploma (postsecondary level)	None	>RM 6000
6	25	Male	First degree	Private sector	RM 3000-RM 4000
7	31	Female	First degree	Private sector	>RM 6000
8	23	Female	First degree	Private sector	>RM 6000
9	30	Male	Postgraduate	None	<RM 1000
10	49	Female	Diploma	Government sector	RM 4000-RM 5000
11	19	Male	Diploma	None	RM 3000-RM 4000
12	40	Male	First degree	Self-employed	RM 5000-RM 6000
13	29	Female	First degree	Government sector	>RM 6000
14	22	Female	Secondary School	Private sector	RM 1000-RM 2000
15	33	Female	Postgraduate	Private sector	>RM 6000
16	32	Male	Postgraduate	Government sector	RM 5000-RM 6000
17	37	Female	Diploma	Private sector	RM 2000-RM 3000
18	31	Female	First degree	Private sector	<RM 1000
19	23	Male	Secondary School	Private sector	RM 4000-RM 5000
20	34	Male	First degree	Private sector	>RM 6000
21	22	Male	First degree	None	RM 2000-RM 3000
22	29	Male	Postgraduate	None	<RM 1000

^a^RM 50=US $11.

In total, 3 main themes were identified and will be discussed in detail in subsequent paragraphs with representative quotes: emotional responses and impacts of health misinformation, approaches used to identify health misinformation, and responses to health misinformation.

### Emotional Responses and Impacts of Encountering Health Misinformation on Social Media

Respondents generally regarded health misinformation as a serious issue with important and potentially harmful consequences. Many reported encountering such misinformation across various social media platforms, including WhatsApp, X, Facebook, and TikTok (ByteDance). The topics described included misleading claims about COVID-19 (eg, consumption of clove water, coconut water, or black pepper water as cures for COVID-19), conspiracy theories regarding health treatments (eg, pharmaceutical companies concealing cancer cures), vaccinations (eg, vaccines causing autism or COVID-19 vaccines containing tracking devices), and pseudoscientific claims related to traditional supplements (eg, turmeric curing all ailments).

Respondents described a variety of emotions when they encountered health misinformation on social media. They felt uncertainty because of the conflicting information being spread, leading to worry as they were unsure which was correct. In addition, when they came across health misinformation on social media, they experienced self-doubt, questioning their own knowledge as the health misinformation conflicted with their own understanding of the health issue at hand:

This is worrying as one person says something and another person says something else. So, we are not sure if it is right or wrong.

Respondent 11

It would get me thinking that whatever I studied, is it wrong?

Respondent 7

They explained that they felt scared as they saw even health care professionals spread health misinformation on social media, especially during the peak of the COVID-19 pandemic when everyone was uncertain about what was happening. This further led to feelings of vulnerability as they were not from a health care background, and with health care professionals themselves believing health misinformation, this put them in a more vulnerable position:

I saw videos with healthcare workers saying that vaccination could make our immune system worse. This is scary for me as if they can be uncertain about it, what about those who are not from a healthcare background?

Respondent 7

Besides that, some respondents described feeling angry as they found the health misinformation spreading on social media to be ridiculous. They further explained that this health misinformation was selling hope to those who were sick, which they felt was very irresponsible. Furthermore, another respondent noted that during the peak of the COVID-19 pandemic, when vaccination was promoted by authorities as a solution, the spread of antivaxxers on social media led to anxiety, as they felt they were exposed to the virus when they came across them in a public area:

Who says coconut water can kill Covid-19? They make me so angry.

Respondent 1

How can this kind of food recover the function of the kidneys? It’s irresponsible to be spreading this information. They are selling hope. Those who are sick are willing to do anything or spend money to get their health back.

Respondent 12

It gives us anxiety when faced with anti-vaxxers, especially at the peak of the pandemic.

Respondent 2

Some respondents described feeling disappointed as they were lied to with promised recovery through miraculous treatment options that failed:

You just feel you’re being lied to, and then you get your hopes down.

Respondent 22

However, a few respondents indicated that misinformation was rare and not serious or significant. This was because they rarely encountered misinformation and had only mild or no effects:

It is rare that there is misinformation at all.

Respondent 18

### Approaches Used to Identify Health Misinformation

Respondents indicated the approaches they used to identify health misinformation, which can be divided into 2 subthemes: message characteristics and the source of the message on social media.

#### Message Characteristics

Respondents examined the characteristics of social media messages to decide whether they were health misinformation. This comprised the message content and layout.

Respondents decided that it could be misinformation when claims were illogical and exaggerated or when the message claimed that the product is a magical cure for every ailment and was too good to be true. In addition, it was potentially misinformation if the tips or treatment suggested were too simple compared with the severity of the illness at hand, whereby the information provided was deemed to be clearly false:

If it is either too good to be true or sounds ridiculous.

Respondent 2

It is not logical, how can this type of herb cure everything?

Respondent 12

Some respondents also expressed doubt about the products being promoted, as they lacked proper dosage guidelines in comparison to medications provided by health care professionals. One respondent highlighted that messages that appear visually unattractive should be investigated further, as official information would be created with much thought and graphics to ensure appropriate dissemination of information to the public. In addition, messages circulated on WhatsApp with the caption *forwarded many times* should be approached with caution:

As most of the time, it is simply forwarded many times. When we see this tag on WhatsApp, it has a high chance of being something that is circulated for the sake of circulating, while nobody actually knows whether it is correct.

Respondent 22

#### Source of Message

##### Overview

Respondents highlighted that health misinformation was commonly encountered on blogs, Facebook, X, and WhatsApp groups. They identified several key factors related to the source of a message when determining whether health information was misinformation. These factors can be grouped into 3 main areas: verifying misinformation through source credibility, which focuses on the objective validation of the source based on institutional backing or official endorsements; social trust and misinformation on social media, which emphasizes the role of perceived trustworthiness and social influence of the message source; and sociodemographic influences on source credibility, which considers how factors such as education level, geographic location, and age shape the perceived credibility of the source.

##### Verifying Misinformation Through Source Credibility

The perceived credibility of the information source was a major factor in identifying health misinformation. Respondents consistently mentioned that health misinformation was often associated with sources that lacked verification or evidence-based backing. Government bodies, such as the Malaysian Ministry of Health, and verified organizations (eg, nongovernmental organizations with blue-ticked social media accounts) were seen as more trustworthy. Messages that contradicted or lacked support from these credible sources were more likely to be considered misinformation. For instance, a lack of official approval or research backing from the Ministry of Health, or promotion of unregistered products, were key indicators that the information could be false. In addition, information not reported by traditional media sources was also deemed as possible misinformation:

Suppose they have at least a blue tick source, I will be more confident as some government pages and NGOs have a blue tick. You know they are verified and that gives a bit more trust factor. But if it is only some small shop that sells health ointments or whatever, like nothing is credible, no information; then for those, I will definitely have to search for extra information.

Respondent 1

Okay, I know it is [misinformation] because I never found the information in the newspaper or news, and the Honorable Minister of Health has never allowed its usage in health.

Respondent 9

##### Social Trust and Misinformation on Social Media

In general, respondents highlighted that health misinformation mainly comes from individuals rather than organizations, and any individual could potentially spread misinformation, including vaccine opponents, family members, friends, celebrities, health care professionals, and business-oriented individuals. Furthermore, those who lack knowledge of how to obtain accurate information were considered potential sources of misinformation. Those spreading misinformation about health were described as having a strong belief in their stance:

They have a lot of followers, it’s like cults. For example, if they post something, and you comment with scientific evidence, they will [continue to] reject because they have been brainwashed.

Respondent 17

They do not know where to find the correct information, so whatever information they get, they feel it is true.

Respondent 20

Respondents highlighted important characteristics related to social trust in the source of a message, which significantly influenced their perception of health misinformation. They noted that individuals who have a strong social media presence and a trustworthy image are more likely to be believed. This trust is particularly important when assessing the credibility of health information. Respondents indicated that individuals with strong educational backgrounds, professional qualifications, or verified social media profiles were seen as more reliable, thus emphasizing the image portrayed on social media being of utmost importance. Conversely, individuals lacking these credentials were viewed as less trustworthy.

##### Sociodemographic Influences on Source Credibility

Respondents also discussed how sociodemographic factors influenced the perceived credibility of sources of health misinformation. Education level was identified as a key factor, with respondents perceiving that people with lower educational backgrounds were more likely to spread misinformation. Geographic location, particularly rural areas, was another significant influence, with respondents believing that people in rural areas had limited access to reliable health information. Age was also mentioned, with older individuals often seen as sources of misinformation, although respondents acknowledged that misinformation could originate from any age group:

Those less privileged, less educated, or [from] rural areas, they tend to share a lot of fake news.

Respondent 13

Especially parents, those in their 50s, share a lot of [such information], but they don’t really know whether it is true or not.

Respondent 22

#### Challenges Associated With Identification of Health Misinformation

Respondents indicated that identifying misinformation becomes difficult when a message possesses certain characteristics. These characteristics included lengthy posts and messages that spread virally. Furthermore, messages that contain anecdotes and testimonials appeared convincing, as they had been tried by many people with positive results. Similarly, messages and videos that feature endorsements from academicians or health care professionals for treatments were also deemed challenging to differentiate. Some respondents perceived that they could trust the message if it went viral. Hence, viral messages can create confusing outcomes for the general public, as some believe that the message is reliable, while others believe that it might be spreading misinformation:

It looks like something genuine because they include anecdotes and testimonials. They include videos like I’ve tried this.... So, when you have these kinds of anecdotes, people are influenced by the power of emotions.

Respondent 2

They quote doctors claiming the latest evidence shows that high cholesterol is normal, safe, and can prolong lifespan. So, the public will believe they can lead a sedentary life and consume a high-fat meal.

Respondent 4

### Responses To Health Misinformation

Responses to health misinformation can be divided into 3 themes: ignoring, verifying health information, and taking action.

#### Ignoring

Many respondents disclosed that they often chose to simply ignore or disregard health misinformation and cited several reasons for this.

Some respondents mentioned that they would ignore the misinformation when they are uncertain about the health topic, if it was controversial, or if it came from unknown strangers. In addition, many respondents believed that they lacked sufficient expertise in health matters to offer informed opinions and, thus, preferred not to risk appearing ignorant on social media. Some were also concerned that challenging the misinformation might lead the perpetrator to question their qualifications, and that could backfire:

I want to fix [correct] it. But the netizens will comment “Who are you to tell this?” So, I leave it alone.

Respondent 10

Because we do not have the knowledge, I am a public person, so we follow those who have studied. When they argue these types of topics, we simply leave it.

Respondent 19

Another reason cited by respondents for ignoring health misinformation was to avoid potential trouble and conflict. They expressed concerns that challenging the perpetrator might lead to cyberbullying or negative reactions. Others expressed feelings of fear or concern about offending or embarrassing the person sharing the misinformation, which led them to refrain from challenging the content, highlighting how emotions, such as empathy, fear, and worry, influenced their decision to remain passive. Furthermore, 1 respondent highlighted that they would ignore the misinformation if there were many supporters for those sharing misinformation:

I will not interfere with them. If the poster [person posting the misinformation] wants to look for trouble with you or the people who follow that person are really supportive of that person, then I am going to be in trouble.

Respondent 22

If there are many people in the group that you do not know and you do not know how they will react, I will just ignore them. It is because I try to avoid conflict and trouble.

Respondent 22

A few respondents mentioned that they would ignore the misinformation when it does not affect them personally. They believed that it would require too much effort to try to correct the misinformation and that they would not be able to make a difference in any case. Some simply believed that someone else would correct it, while others felt that it was the responsibility of the government to take corrective actions:

A lot of people seem to believe that [misinformation]. So, you cannot really change their minds.

Respondent 1

It is a mentality of how much difference am I going to make?

Respondent 7

In addition, 1 respondent believed that the perpetrator’s opinion should be respected as a form of free expression allowed on social media. They added that condemning people for their opinions is not an acceptable practice.

#### Verifying Health Information

Most respondents described verifying health-related information that they come across on social media and shared various reasons and methods they used to verify the information. Verification usually involves checking both the content of the information and the background of the individual or organization sharing it. Many indicated the importance of verification, even if the source of the information is a health care professional or family member:

When they post something, I will search about it first, even if my family members share something.

Respondent 14

Respondents reported that they were more likely to check information if it pertained to a serious issue that could potentially affect them. They also mentioned that they would verify information if they were uncertain about it, for instance, if the information was difficult to understand or if they had never heard about it before. They were also more likely to verify information that came from fewer sources or sources with questionable reliability and trustworthiness. In addition, respondents said that they would verify information if conflicting information was presented or if they were considering spending money, for example, on a product advertised:

I will check if I never heard about it before.

Respondent 21

I would look for more information if it affects me or I have to spend money for it.

Respondent 3

When they post, usually I check their backup sources, from where they share their information...At least if there is research or link to reliable sources. I do not like it when they share something not to be done, without any evidence.

Respondent 13

When evaluating the credibility of health-related information, respondents typically used multiple sources, including social media, health care professionals, health organizations, traditional media, family members, acquaintances, websites, and studies. Some respondents mentioned that they could identify false information because the correct information had already gone viral. If the respondents still had doubts, they simply visited a doctor to clarify the information. The respondents emphasized the importance of being vigilant and having critical thinking skills to distinguish between true and false information:

I will read a lot more than just Facebook. Maybe I will Google first and then read. I will read the type of feedback provided by other people.

Respondent 17

I will check with doctor friends, pharmacists, and nurses.

Respondent 9

#### Taking Action

This theme relates to respondents describing the actions that they would take to address health misinformation on social media. If respondents were to take action, it would be either to report, block, or correct misinformation. Respondents also discussed who should be responsible for correcting the health misinformation.

Many respondents mentioned that they would report the misinformation despite perceiving limited regulations on social media. The reporting actions included notifying relevant social media administrators; law enforcement agencies, such as the National Pharmaceutical Regulatory Agency; and the Ministry of Health. Interestingly, 1 respondent said that they would tag the police department. These efforts were aimed at ensuring the misinformation was removed from the respective social media platforms:

If I am sure it is wrong, there is a button where you can press report. Yeah, I will just do that.

Respondent 14

Some respondents indicated that they did not want to ruin their social media page with content that they disagreed with. Thus, they would either mute or block the offender’s account, while others deleted those who posted misinformation from groups.

Many respondents believed that correcting misinformation is important and expressed their willingness to do so if they encounter false information. However, respondents mentioned that they were more likely to provide corrections in certain situations. Generally, they were inclined to correct misinformation if they felt there could be no conflict or direct confrontation as a result. For example, if they knew the person posting misinformation, they would feel comfortable providing corrections, driven by a sense of familiarity and trust. They felt safe correcting misinformation through personal WhatsApp messages or group chats if they were familiar with everyone in the group. In addition, respondents described feeling more confident in correcting misinformation if they were knowledgeable about the topic and if others had already corrected it before them. They were also more motivated to provide corrections and encourage others to verify the information source if they believed it could affect their family members, indicating how emotional factors such as care and responsibility can motivate actions against misinformation:

If there are people close to me, for example a group of five people that I am close to, close friends, then it will be easy for me to correct them.

Respondent 22

If there are others who have commented it is wrong, then I will comment. Otherwise, I will not.

Respondent 14

If it affects my family, I would shut it down immediately. I will tell them that they would want to check the sources. But when it comes to other people, I mostly would not budge [react].

Respondent 7

When correcting misinformation, respondents provided justifications for why the information was false and sought to persuade using data and facts. A few respondents also mentioned using general terms and simple language, similar to that used by those disseminating false information. In addition, respondents shared information from sources deemed reliable, including social media sources with blue verification ticks, to correct misinformation:

Try to speak things in terms of their perspective, like put yourself in their shoes and try to speak in their language. However, if things fail, then try to convince with facts and figures.

Respondent 2

Take the evidence from the blue tick source.

Respondent 21

Respondents described experiencing mixed outcomes when correcting misinformation. Some family members were thankful and believed the corrected information, while others deleted the false information post. Unfortunately, some respondents faced reprimand and subsequently chose to disregard it. Despite the possibility of disbelief from others, they believed it was their duty to educate them regarding the truth. However, some respondents reported that their comments went unanswered, and in some cases, the offenders shared even more questionable links. Nevertheless, it was emphasized that respondents had done their part in correcting the inaccurate information.

#### Who Should Correct Health Misinformation on Social Media

The respondents believed that the government should take action against the spread of misinformation. In addition, it was suggested that the government should adopt measures to educate the general public about the dangers of misinformation and to be cautious before sharing health information on social media. Furthermore, respondents proposed enforcing legal provisions and regulations against those spreading health misinformation. An interesting suggestion was made that the Ministry of Health could use nudges on social media, such as “Are you sure about this?” to encourage the general public to consider the validity of health information before sharing it:

I hope the government of Malaysia will tighten the law for those who like to spread wrong information, because if they do not, they will keep doing it.

Respondent 19

For example, Ministry of Health can comment “Are you sure this fact is true?” and people will be thinking, okay this may not be true because Ministry of Health commented.

Respondent 22

Respondents also recommended that the Ministry of Health establish a multidisciplinary team dedicated to verifying and correcting misinformation on social media. They also highlighted an opportunity to improve accessibility by providing government health information in languages beyond English and Malay to better accommodate a multicultural population. Furthermore, participants emphasized the importance of providing timely and transparent explanations for changes in health-related information, as observed during the COVID-19 pandemic, when frequent updates were necessary because of emerging evidence:

They should have a team or department that targets or even tracks all these rumors being spread around.

Respondent 7

I wish they would have more languages. At this point, it is mostly in English and Malay, and there are many elderly people who do not understand.

Respondent 7

During the Covid-19 pandemic, initially they [government] provided a recommendation [on] vaccines...then they provided different information...but they never explained whether the vaccines were really effective.

Respondent 6

The public may not understand that knowledge is progressive. Initially, the government recommended two doses to achieve herd immunity, but when cases were rising, they introduced booster doses. The public will hold on to the recommendations made two years ago.

Respondent 16

In addition to the government, respondents also believed that health care professionals have a responsibility to combat misinformation. It was suggested that health care professionals should be more proactive in correcting misinformation on social media and consider reaching out to populations considered vulnerable:

Healthcare workers such as doctors and pharmacists should correct people’s understanding on medication. If more people share on social media, it is better so that the wrong and true information are in the same quantity.

Respondent 13

When doctors post on health information, they have to think how to reach those susceptible to this [misinformation].

Respondent 13

## Discussion

### Principal Findings

The spread of health misinformation through social media platforms has led to uncertainty and evokes a range of emotional responses in those who encounter it. This study provides insights into the various approaches used by the general public to identify health misinformation on social media as well as the reasons and motivations behind their responses to it. In addition, several challenges faced by the general public in identifying and addressing misinformation were identified.

The first phase of TMIM describes the uncertainty caused by health misinformation. Our study reflects this, showing that misinformation spread through social media platforms led to uncertainties and triggered emotional responses, such as worry, fear, self-doubt, anger, feelings of vulnerability, disappointment, and anxiety. Studies in other countries also showed this, where misinformation in South Africa caused panic, confusion, and anxiety [53]. Similarly, studies in Jordan and Spain reported elevated levels of anxiety in response to health misinformation [13,54]. These emotions significantly influence how individuals process and respond to misleading health information. Higher levels of anxiety and fear, for example, are associated with increased belief and willingness to share misinformation [55,56]. Anger, which often arises when users feel deceived by false claims, also contributes to intuitive actions and the further spread of misinformation [57,58]. Fear and anxiety tend to intensify when misinformation relates to health threats, increasing concerns and feelings of vulnerability over personal and general public health [59]. Uncertainty and self-doubt emerge when users encounter conflicting information, leading to cognitive dissonance that hinders informed decision-making [60]. Such emotional distress can lead to a general feeling of disappointment, especially when people realize that they have been misled by trusted sources or when accurate information is overshadowed by misinformation [57]. The cumulative effect of these emotions can hinder effective health decision-making and reduce trust in health information sources [12-15]. This may influence the evaluation phase, where individuals analyze and attempt to identify health misinformation on social media platforms.

Previous research has identified both internal and external factors that individuals use to determine the credibility of the information [61-63]. Internal factors include elements such as the source of the message, message characteristics, and individual personality traits. By contrast, external factors include institutional sources or interpersonal networks, such as verification from family, friends, and trusted institutions. This study also described the internal and external factors used by respondents to assess credibility and the challenges faced in identifying misinformation. One significant internal factor is the source of the message. Trust in government and health care professionals emerged as an important component, highlighting how credibility is tied to recognized, authoritative sources. This aligns with the MAIN (modality, agency, interactivity, and navigability) model, which suggests that technological aspects of digital media can influence credibility judgments, particularly when information comes from official authorities [64]. Supporting this, a study conducted in Malaysia found that respondents mainly selected the Ministry of Health as their preferred source of health information [65]. This trust may stem from cultural norms in Malaysia, where the conservative Asian context fosters greater respect for figures of authority [66]. Furthermore, respondents believed that the government has a critical role to play in combating this problem by implementing regulations and awareness campaigns. Beyond institutional sources, respondents also expressed trust in messages from those they believed to have trustworthy reputations and relevant educational backgrounds. This was also similar in India, with respondents placing trust in messages from local government representatives and community health workers deemed to have a wide knowledge of the area [62].

In contrast to previous studies conducted in Malaysia and the United States, where most respondents were unable to evaluate the accuracy of health-related information, our study identified specific characteristics of messages that respondents associated with misinformation [67,68]. Respondents pointed out that exaggerated or illogical claims, such as those promising miracle cures, were indicative of misinformation. This was similarly seen in a survey study in Austria, where exaggerated claims were met with skepticism [69]. Conversely, social media messages that were visually appealing were often perceived as more credible by respondents. Research has indicated that visual design plays a role in influencing people’s judgments about the credibility of health information found on the web [70]. This may be because of the attractive design of these messages, such as well-crafted infographics, which suggest that effort and thought have been invested in creating them, which in turn increases their trustworthiness [71].

Correction directed to those sharing the misinformation and individuals looking at misinformation was considered a strategy for combating health misinformation [30]. A content analysis of monkeypox on Instagram found that one-third of the content was debunked, with social media users actively correcting the misinformation [72]. It was interesting that in our study, many respondents chose to ignore misinformation, citing avoidance of conflict and perceived futility in correcting it. This behavior aligns with findings from another Malaysian study, which showed that most respondents tended to ignore fake news during the COVID-19 pandemic [68]. Such tendencies may be influenced by cultural norms, as previous research has found that Malaysians often prefer to avoid conflicts [73]. Nevertheless, this finding is also consistent with other studies [62,74-76], including a UK study that found avoidance of conflict to be a major barrier in preventing the correction of COVID-19 misinformation [76]. However, despite these challenges, some respondents in our study were willing to take action when their loved ones were affected or when they had a close relationship with those posting misinformation. This could be beneficial, as the literature suggests that correction by a close tie could be successful [77]. In addition, corrections that focus on collective interests have been shown to work better [78].

Respondents also were more inclined to correct misinformation when they felt that they knew the topic well. This is supported by previous studies where health care workers themselves face challenges in correcting misinformation because of various factors, such as the belief that there would be no improvement after correction; lack of time to address the issue; fear of retaliation from those posting misinformation; and a lack of support, such as social media training, to handle these situations [79]. They opted to correct misinformation only if it was important to them or if the offender was someone close to them [74]. Furthermore, an experimental study among health experts in China showed that they were willing to correct health misinformation when the perceived threat to readers was severe, when they believed that they had the necessary skills to correct it, and when they wanted to maintain a reputation of kindness toward others [80]. This aligns with a study in India where respondents chose not to correct misinformation if they believed it would not cause harm [62].

### Suggestions for Interventions to Tackle Health Misinformation

Multifaceted interventions involving multiple stakeholders, including government, health care providers, researchers, academicians, and social media administrators, are needed to effectively address health misinformation on social media. Previous studies have recommended measures to combat health misinformation in Malaysia, such as legal action against offenders, the establishment of fact-checking portals, and the continuous health information dissemination to the general public [81]. The findings from this study suggest additional measures that can be implemented to further address the spread of misinformation. Respondents identified several challenges in identifying misinformation, including those presented with anecdotes or testimonials. It has been acknowledged that misinformation can spread when compelling anecdotes are presented and data are misrepresented with fake experts [82]. Studies also revealed that correct information and misinformation both contained anecdotes, which explains why the general public may be confused in this regard [83]. This highlights the need for digital literacy training on how to search for, identify, verify, and share credible health information on social media, as indicated by other studies [84-87]. Improvement in digital literacy skills was seen to improve recognition of the quality of health information on the web [88-91]. Educational institutions should also focus on increasing awareness regarding the identification of health misinformation on social media [92-94]. This was shown to lead to fact-checking information before sharing [95].‬‬‬‬‬‬‬‬‬‬‬‬‬‬‬‬‬‬‬‬‬‬‬‬‬‬‬‬‬‬‬‬‬‬‬‬‬‬‬‬‬‬‬‬‬‬‬‬‬‬

In addition, messages that are viral need to be monitored, as they tend to cause confusion regarding credibility, as indicated by respondents in this study. Another study supports this, showing that among African American older adults, respondents were more likely to believe a message if they saw the information multiple times [96]. Furthermore, other studies have shown that health misinformation with a greater number of likes is often perceived as more credible, as it was viewed as social endorsements [18-20]. One potential strategy is to assemble a team of health experts to correct misinformation on social media and highlight the truth, with the support of multiple experts to prevent cyberbullying toward them [79,86,97]. In addition, health care workers can address health misinformation by correcting it when they encounter it with patients at clinics or in hospital settings. This can be done by educating the patients and encouraging 2-way communication between patients and health care workers [98,99]. Moreover, the government could focus on “prebunking” by addressing potential areas that could pose challenges in differentiating misinformation before it spreads [100,101]. As “prebunking” effect is limited, this strategy should be combined with other efforts to enhance its effectiveness in combating misinformation [102].

Access to multilingual health information has also been identified as a need, with potential implications for health equity. The WHO has acknowledged multilingualism as an area to improve equality in health information dissemination [103]. However, further investigation is required to determine the extent of implementation across countries and to assess the need for additional languages in health information dissemination to effectively combat misinformation.

### Limitations

This study offers insights into public perceptions of health misinformation on social media in a Southeast Asian country, a region with limited research on the topic. The challenges described can be used to develop interventions to address the issue of health misinformation on social media. Nevertheless, this study has some limitations that need to be considered, including the possibility of sampling biases and social desirability biases. Although efforts were made to include a wide range of respondents from various sociodemographic backgrounds, it is possible that certain groups may have been missed, such as those with lower educational backgrounds or older respondents. Further quantitative research is recommended to better understand how different population groups identify and respond to health misinformation. The development and validation of tools to measure this, such as survey-based approaches to validate identified themes, would be valuable for enabling cross-country comparisons regarding responses to health misinformation.

### Conclusions

The characteristics of a message and its source on social media are important factors that the public considers when identifying health misinformation. Various reasons and circumstances may affect individual responses toward health misinformation, which range from ignoring it to verifying health information and adopting measures to correct it. Digital literacy training may be useful in addressing the challenges faced by the public in identifying and responding to health misinformation. This study also highlights the need to further investigate populations considered vulnerable or at risk of health misinformation on social media and the factors influencing responses toward health misinformation, which will allow the development of targeted intervention strategies.

## Abbreviations

TMIM: theory of motivated information management
WHO: World Health Organization

## Data Availability

The datasets generated or analyzed during this study are not publicly available due to ethical concerns but are available from the corresponding author on reasonable request.
